# Role of nurses in the early recognition of sepsis

**DOI:** 10.1186/cc10171

**Published:** 2011-06-22

**Authors:** P Padilha, B Almeida, BC Derico, FCM Elmiro, MFF Jesus, VL Sousa

**Affiliations:** 1 Centro Universitário Ítalo-Brasileiro, São Paulo - SP, Brazil

## Introduction

Sepsis is considered one of the most challenging diseases of all time [1]. During many years the concept of sepsis was not the same inside the medical court, which resulted in a heterogeneous population [2]. Its incidence has been growing dramatically over the past decades, having advanced age of patients, increase of invasive procedures, frequent use of immunosuppressive drugs and the increase of infections caused by multiresistant bacteria as the main contributors [3]. Nurses have an important role in early recognition of sepsis.

## Objective

We investigated whether nurses are able to early recognize signals and symptoms of sepsis.

## Methods

The methodological strategy was quantitative, exploratory and multicentric, with four small private hospitals involved. Thirty nurses working for medical-surgical clinic, semi-intensive, intensive and emergency units participated in the survey.

## Results

Only 23.3% of nurses considered variation in leukocytes, cardiac and respiratory frequency and axillary temperature as classifying sepsis clinical signals. In one case with sepsis signals, only 10% of answers were correct. When trying to establish a differentiation pattern among sepsis stages, a new case was developed highlighting severe sepsis, showing 36.6% of right answers, making it clear that there is a confusion facing this syndrome, where 10% chose sepsis, 26.6% septic shock and 26.6% infection caused by a surgical wound. Only 30% of the nurses pointed out that treatment is effective within hours of its recognition. In a final question 70% affirmed that it is important to recognize sepsis early.

## Conclusion

The study showed that there are difficulties on the part of nurses in recognition of sepsis. With the present results, it can be concluded that the development of nursing care protocols with the early recognition of sepsis signals by the nurse can help the patient's recovery. Training of nurses working in an ICU, and their team as a whole, can help to reduce deaths in hospitals, improving the assistance and making patients' permanence in ICUs shorter, which can not only benefit patients but can also lead to a reduction in costs for the institution.
